# Creating a sport and exercise medicine undergraduate syllabus: a delphi study

**DOI:** 10.1186/s12909-023-04139-x

**Published:** 2023-03-23

**Authors:** Dane Vishnubala, Adil Iqbal, Katherine Marino, Tej Pandya, David Salman, Andy Pringle, Camilla Nykjaer, Peter Bazira, David Eastwood, Gabrielle Finn

**Affiliations:** 1 Hull York Medical School, York, UK; 2 University of Leeds, Leeds, UK; 3 Royal Stoke University Hospital, Stoke-on-Trent, UK; 4 Royal Bolton Hospital, Minerva Road, Farnworth, BL4OJR UK; 5 School of Public Health/MSK lab, Imperial College London, London, UK; 6 Sport Outdoor and Exercise Science, University of Derby, Derby, UK; 7 Faculty of Biological Sciences, University of Leeds, Leeds, UK; 8 University of Manchester, Manchester, UK; 9 University of York, Heslington, York YO105DD UK

**Keywords:** Medical education, Sport and exercise medicine, Undergraduate

## Abstract

**Background:**

Sport and Exercise Medicine (SEM) is a growing speciality in the United Kingdom (UK). This growth has not been replicated in SEM teaching at an undergraduate level and SEM-related topics in schools of medicine in the UK are under-represented. As SEM continues to develop as a specialty it is important to consider how it is embedded at all levels of training. The aim of this project was to establish a consensus on SEM-related skills and knowledge relevant for undergraduate medical students in the UK, ultimately creating a curriculum of learning objectives (LOs).

**Methods:**

A modified Delphi survey was utilised to seek consensus on LOs suitable for incorporation into UK medical school curricula. An expert panel with adequate knowledge in the field was recruited. The initial curriculum was created by the research team using already established postgraduate SEM curricula. All learning objectives were sent to the expert panel for opinions in phases. Levels of agreement and comments made by the expert panel were reviewed after each phase until a consensus on each learning objective was made.

**Results:**

The expert panel was made up of 45 individuals, with 35 also completing phase 2 (78% retention rate). The initial curriculum contained 58 learning objectives separated into 9 themes. In phase 1 31% (18/58) were accepted outright, 48% (28/58) were altered and 19% (11/58) were rejected. Two additional learning objectives were added. Of the 49 LOs included in phase 2, 98% (48/49) were accepted. The final curriculum was made up of 9 sub-themes and 48 LOs.

**Conclusion:**

Sport and Exercise Medicine is a broad ranging and rapidly growing speciality. It is important to establish SEM education in all levels of medical education, including undergraduate level. This is the first published version of a Delphi SEM curriculum for undergraduate medical teaching.

**Supplementary Information:**

The online version contains supplementary material available at 10.1186/s12909-023-04139-x.

## Background

Sport and Exercise Medicine (SEM) is a growing medical speciality in the United Kingdom (UK) and worldwide. SEM is a medical speciality that includes team medicine, exercise medicine and musculoskeletal medicine. SEM could be an important medical speciality to improving the health status and quality of life of patients by both increasing and maintaining physical activity levels. Physical inactivity is a leading cause of disease burden and an important modifiable risk factor alongside smoking. [1] The past few years have seen position statements, the creation of multiple postgraduate SEM courses, and an international Delphi study to create a curriculum for SEM practitioners. [1–3] However, this growth has not been replicated in SEM teaching at an undergraduate level, and SEM-related topics in schools of medicine in the UK are under-represented. [4–6] The General Medical Council’s “Outcomes for Graduates” and Health Education England’s “Future Doctors” reference the growing need for doctors to be skilled in providing tailored exercise medicine advice to patients, and in demonstrating core musculoskeletal skills. [7,8] Most medical specialties currently have a syllabus to which they expect medical students to achieve outcomes by the end of their undergraduate career.

Worldwide, the integration of sport and exercise medicine teaching into undergraduate curricula is also an issue. In the USA, Asif et al. (2022) conducted a Delphi study for an exercise medicine core curriculum because medical trainees in the USA were found to receive relatively few hours of teaching [9]. This global training issue also affects the Middle East and Europe. [10,11]

As SEM continues to develop as a specialty it is important to consider how it is embedded at all levels of training. In the UK, it is common practice by the majority of royal colleges to produce an undergraduate curriculum. Creating and encouraging the use of that curriculum by medical schools could be useful to ensuring exposure and interest to the speciality. SEM is a relatively young specialty, having been formally established in 2005, and no equivalent undergraduate curriculum has been established to date. [12] With this in mind, the aim of this project was to establish a consensus on SEM-related skills and knowledge relevant for undergraduate medical students in the UK, ultimately creating a curriculum of learning objectives (LOs). This will act as a guide for the teaching of SEM at undergraduate level and standardise undergraduate SEM teaching throughout the UK. The majority of medical schools do not follow, nor need to follow published royal college undergraduate curriculums, their priority it to ensure they meet GMC outcomes for graduates. Specific learning outcomes are usually created by the medical school and for the majority of specialities this is entirely appropriate given they are large specialities found in all areas. SEM however, is a small speciality with only 173 Consultant doctors on the GMC specialist register based in a narrow range of locations and with currently a limited but growing NHS presence. [13] It is therefore unlikely that all medical schools have access to Sport and Exercise Medicine Clinicians. It is hoped this consensus of LOs will encourage medical schools to increase the amount of SEM teaching incorporated into medical school curricula.

## Methods

### Study Design

A modified Delphi survey was utilised to seek consensus on LOs suitable for incorporation into UK medical school curricula. The original delphi method was developed by Dalkey and Helmer and is an iterative process designed to determine consensus through exposing the expert panel to multiple iterations of data, in this case learning outcomes. [14,15] There are a variety of observed ways of delivering a delphi however its overall distinct features including the use of an expert panel and a round based, iterative approach. Delphi methodology is used extensively in curriculum development. [16] This delphi was defined as modified and therefore variant methodology due to the creation of the draft curriculum by the research group rather than the use of the expert group to create the original curriculum. [15] This methology was used to avoid multiple rounds and therefore the risk of poor response rates with progressive rounds. [17] This study involved the creation of the original draft curriculum by the research team, which was put through 2 rounds of review by the expert panel before being finalised by the research team. In keeping with the principles of Delphi methodology, contributions given by the expert panel were kept anonymous to the research group throughout the process. [18] The methodology used in this study was also used for a Delphi study conducted by members of the research team for postgraduate SEM curricula, the same expert panel was utilised for both studies. [19].

### Establishing the research group

The research group was made up of DV, AI, KM, TP, DS, AP, CN, PB and GF. This group was formed to incorporate individuals with experience in a wide variety of related topics including medical education, delivering SEM education, experience undertaking and/or teaching on undergraduate medical degrees, SEM Masters courses and Delphi methodology. In addition, individuals were included due to their experience with the Undergraduate Sport and Exercise Medicine Society (USEMS) and their interest in the specialty of SEM. USEMS is a UK based, non-profit society aimed at promoting the specialty of SEM for undergraduates. [20] All decisions regarding content were finalised by the research group such as reviewing and amending learning outcomes based on comments from the expert panel following the first round.

### Expert Delphi Panel

Individuals suitable for joining the expert panels are defined as individuals with knowledge and experience in the subject area. [21,22] All members of the British Association of Sport and Exercise Medicine (BASEM) and the Faculty of Sport and Exercise Medicine (FSEM) were emailed invitations to express interest in joining this expert panel. Invitations to submit interest were also shared on Twitter by the research group.

Individuals expressing interest in joining the expert panel were asked demographic information and questions selected by the research panel to determine eligibility. The eligibility criteria were selected to ensure the expert panel was made up of individuals with adequate knowledge in the field of SEM. It was not determined whether the participants had prior experience of writing LOs.

The eligibility criteria used consisted of:

- Doctors that have completed their Foundation Training.
- Achieved a higher qualification in SEM: specifically, either a SEM masters degree/diploma, or membership/fellowship of the FSEM (MFSEM/FFSEM).
- Graduated more than 5 years prior to the start of the study.
- Working in the United Kingdom at the time of the study.

Individuals that did not meet the eligibility criteria were removed by the research group. A panel size of at least 30 was aimed for as the quality of a Delphi study has previously not been found to be improved by a panel size greater than 30. [18,23]

### Development of the initial curriculum

**Fig. 1:**
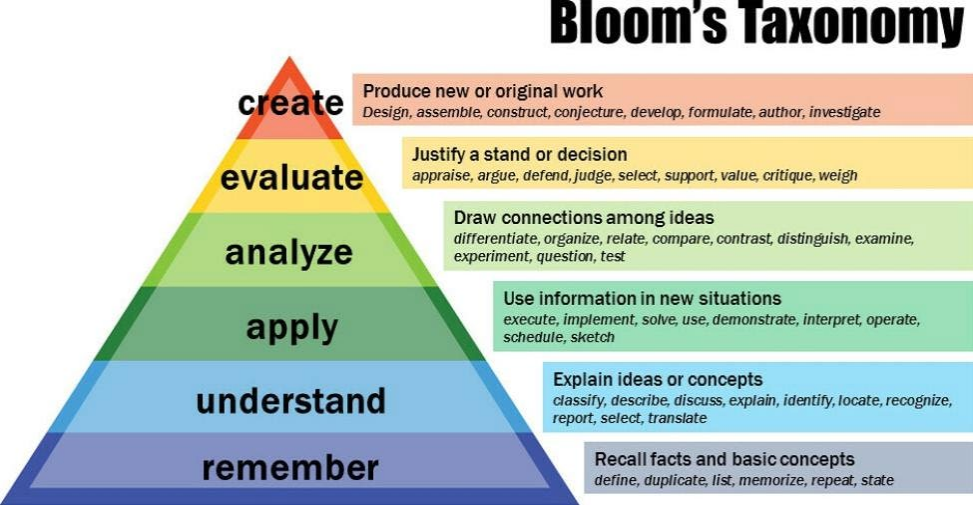
Bloom’s taxonomy. Level 1 is knowledge to level 6 which is creation. Image used within rules of license (creative commons attribution- sharealike license). Taken from: https://commons.wikimedia.org/wiki/File:Bloom%27s_Revised_Taxonomy.jpg

A documentary analysis was performed and LOs included in previously published UK-based SEM specialty training and SEM Masters curricula were combined by the research group. Each learning outcome was then discussed by the research group at a meeting. Learning outcomes that were duplicated or deemed inappropriate for an undergraduate medical student were either removed or amended based on research group consensus. Following the research group review and consensus an initial draft of the curriculum was created. [2,23,24,25] The LOs in these curriculums were grouped into relevant themes as determined by the research group. All LOs were reviewed by the research group before being amended or removed if required. Bloom’s taxonomy was used to establish an appropriate level for undergraduate medical students as agreed by the research group (Fig. 1). [26] Reducing the Bloom’s taxonomy level was the main reason for amending a learning outcome at the draft curriculum phase by the research group. The Bloom’s taxonomy levels used are given in Fig. 1.

### Procedure

Google Forms (Google Inc. USA) was used to create the initial survey for those wishing to express interest in joining the expert group, and demographic information was collected via this form. Qualtrics software was used to create the electronic surveys for the Delphi. [27] This survey was emailed to all individuals deemed eligible to join the expert panel. Participant information sheets were emailed to all eligible individuals. Written informed consent to participation was gained by all members of the expert panel via a mandatory question asked at the start of the survey. The survey instructions stated clearly that experts should consider the relevance of individual LOs for medical student level when answering the survey.

Data were collected from October 2020 to November 2020. Members of the expert panel were given 12 days to respond to each phase of the Delphi. A system-generated email was sent after day 8 and day 10 to non-responders to act as reminders. In addition, members received a text message if no response had been received by day 10. The research group aimed for the response rate to not fall below 70% as expert panel engagement is vital for Delphi studies. [28].

Phase 1: First review of learning objectives.

Members of the expert panel were asked to either accept, reject or modify each LO in the proposed curriculum. To ensure standardisation of approach, the expert panel were asked to focus not only on the content or topic conveyed in the learning outcome but also on the level of Bloom’s taxonomy used. An explanation of Blooms taxonomy as well as links to further resources were provided to the expert panel. A consensus of opinion was defined by 75% agreement, as reported previously by Keeney et al. [18] Ranges from 70 to 100% have been reported in previous literature as appropriate for consensus. [19,29] Participants had the option of providing anonymous comments after reviewing each LO. For each LO the percentage of agreement was calculated and any anonymous comments regarding that LO were reviewed by the research group. All comments for all LOs were reviewed regardless of the level of agreement. The research group then accepted, rejected or altered each LO depending on the responses given to create a second draft of the curriculum. If a level of agreement of 75% or above was received with no comments given, the LO was accepted outright with no further need for review. If comments were given, the research group discussed the comments and, if appropriate, amended the LO and included it in the second draft of the curriculum for further review. If the level of agreement was below 75% and no comments were given, the LO was rejected. If comments were given, the research group reviewed the comments and, when appropriate, amended the LOs and included them again in the second draft of the curriculum for further review.

Phase 2: Second Review of learning objectives.

The second draft of the curriculum, based on responses given in phase 1, was sent via email only to members of the expert panel that had completed phase 1. In phase 2 members of the expert panel were asked to either accept or reject each LO and again there was the option to provide anonymous comments this time after review of each theme rather than each LO. The LOs accepted outright in phase 1 did not require a response but were included for reference. Again, percentages of agreements and comments were reviewed by the research team and decisions were made to accept or reject each LO. A level of agreement of 75% of above was again utilised. Phase 2 would be repeated until a final consensus on each LO was reached.

## Results

### The initial proposed curriculum

There were 58 LOs collated from prior SEM syllabi. These were grouped into 9 core themes by the research group.

### The Expert Panel

Of the 94 people who expressed interest in being on the expert panel, 48% (45/94) met the eligibility criteria. The reasons for non-eligibility included having worked as a doctor for less than 5 years (n = 19), not holding a SEM MSc/Postgraduate Diploma/FFSEM/MFSEM (n = 17) and not being based in the UK (n = 13). The expert panel was made up of 20 SEM consultants, 4 orthopaedic consultants, 1 rheumatology consultant, 17 GPs, 11 SEM registrars and 14 doctors that did not specify their training or job role but did confirm that they had been a doctor for more than 5 years. Thirty-eight individuals (82%) stated they had experience of teaching medical students.

### Phase 1

In phase 1 of the study there was a 100% (45/45) response rate from the expert panel. 31% (18/58) of LOs were accepted without need for alteration and 48% (28/58) were altered in some way. The reasons for alterations are given in Table 1. Nine LOs were altered for more than one reason. Regarding the 8 alterations made to the Bloom’s taxonomy level, 6 (75%) were kept at the level but alterations were made to utilise a more appropriate word from the same taxonomy level, and 2 (25%) were moved up one level.

**Table 1:** **Alterations.** The reasons for alterations to learning objectives after phase 1. Eleven LOs (19%) were rejected and removed due to either lack of relevance to undergraduate level (n = 7) or overlap with other LOs in the curriculum (n = 4). The objectives removed for being too high level, and examples of quotes from the expert panel supporting their removal, are given in Table 2. The objectives removed due to overlap are given in Table 3 alongside the remaining LOs that they overlapped with

Reasons for alteration	Number of learning objectives altered
Spelling and grammar (including re-wording)	14
Alteration to Bloom taxonomy level	8
Objective made more specific	15

**Table 2:** **Removed LOs**. The LOs removed after phase 1 and the comments given by the expert panel that were reviewed by the research group and justify the removal

Learning objective (LO) removed	Expert panel quotes supporting removal
Outline neurological issues in relation to exercise	*There is a limited time within which an understanding of all these issues isn’t able to be achieved and is covered in the wider curriculum (Participant 21)*
Outline renal and urogenital issues in relation to exercise	*Not sure how this could tie in to an UG syllabus (Participant 37)*
Outline ENT issues in relation to exercise	*ENT is too niche (Participant 16)*
Outline basic biomechanics in relation to different sporting and exercise activities and in the context of injury	*Huge area - think unnecessary when need to cover the basics in medicine (Participant 21)*
Outline the role of WADA and UKAD	*Not sure this necessarily needs to be in curriculum at UG level as PG level (Participant 43)*
Describe the on-field and emergency assessment and management of sports injuries and medical conditions	*Pre-hospital care generally not taught at UG level* *(Participant 37)*
Discuss the following in relation to SEM: eye and ENT emergencies	*Relatively small topic and could be integrated with other topics (Participant 16)*

**Table 3:** **Overlapping LOs.** The LOs removed after phase 1 due to overlap with other learning objectives are given on the left. The related learning objective(s) that remained are on the right

Learning objective removed	Remaining learning objective(s) justifying removal
Describe general pathology of the musculoskeletal system.	Relate musculoskeletal anatomical knowledge to common conditions and presentations. Outline the principles of tissue injury and repair in the musculoskeletal system.
Describe the findings of common radiological investigations.	Recognise the indications for common radiological investigations.
Describe the findings of radiological and other relevant investigations.	Recognise the indications for common radiological investigations.
Discuss the following in relation to SEM: Cardiorespiratory arrest.	Recognise the role of pre-hospital care in sport and physical activity. Demonstrate basic life support in a simulated environment.

After review and discussion by the research group 2 additional learning objectives were added: ‘Outline the pharmacological management of acute pain in musculoskeletal conditions in sport and physical activity’ and ‘Identify common adult musculoskeletal conditions’. This was due to it being noted that chronic pain was mentioned in other learning objectives, but the management of acute pain was not covered by any learning objectives and there being no learning objectives specifically including adult musculoskeletal conditions. With the removal of 11 learning objectives and 2 learning objectives added, a total of 49 learning objectives were included in the curriculum for phase 2.

### Phase 2

Of the 45 members of the expert panel that completed phase 1, 78% (35/45) also completed phase 2. 98% (48/49) of LOs were accepted in phase 2 of the study, with these objectives achieving over 75% acceptance. Table 4 gives the level of acceptance for each LO following phase 2. The LO that did not reach 75% agreement was ‘Outline haematological changes and responses to physical activity’ (LO 2f in Table 3). Ten LOs needed to be altered but, as these were all minor grammar and spelling changes, it was felt that there was no need for any further phases. The final curriculum was made up of 9 sub-themes and 48 LOs. Table 5 shows the finalised sub-themes and number of objectives in each sub-theme. The full version of the agreed curriculum can be found in the supplementary material. In the final curriculum, 54% (26/48) of the LOs were in level 1 (knowledge) of Bloom’s taxonomy, 42% (20/48) were in level 2 and the remaining 4% (2/48) were in level 3.

**Table 4:**
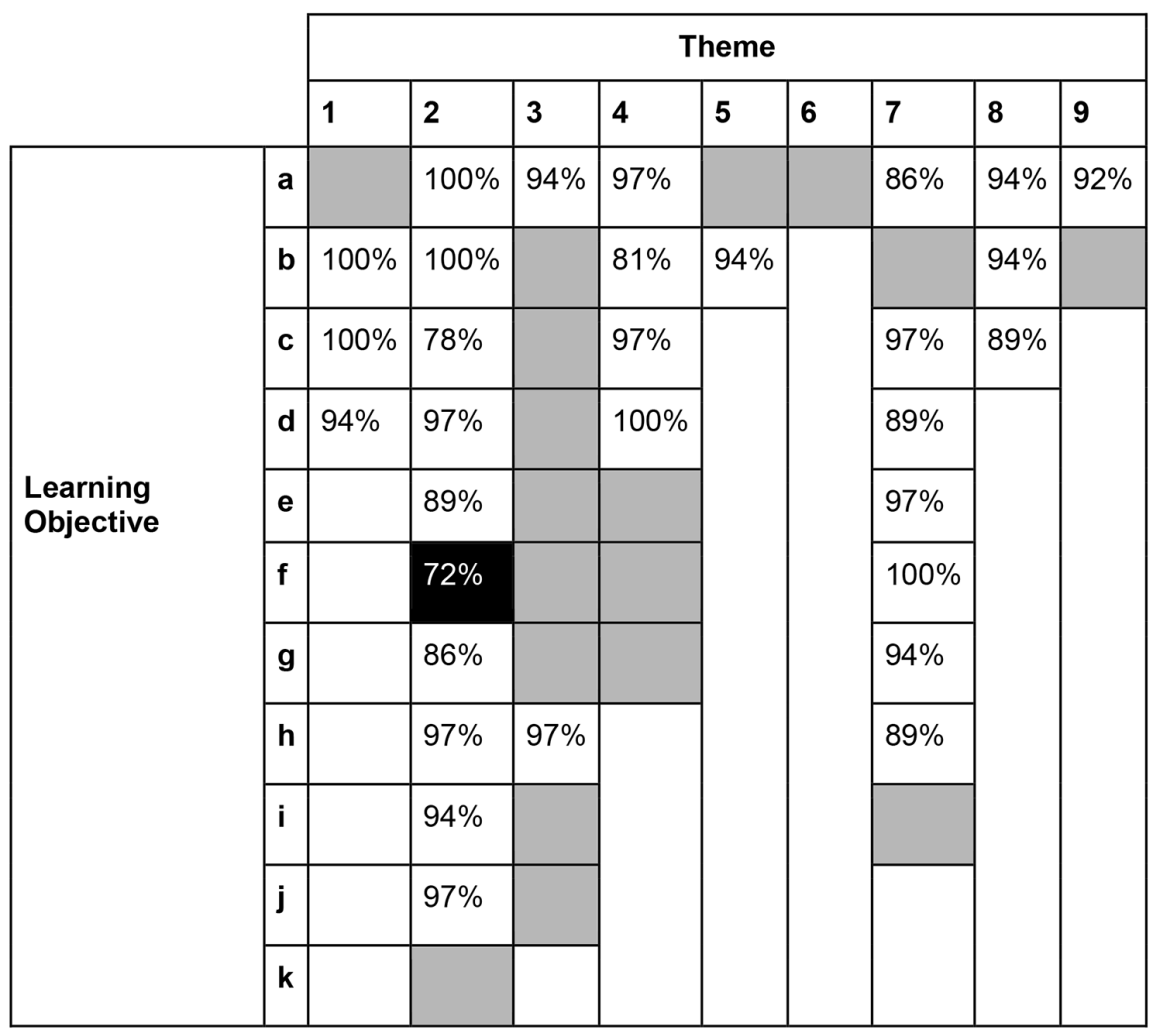
**Levels of acceptance.** The percentage (%) level of acceptance for each learning objective after phase 2. Greyed out boxes indicate the learning objective was accepted outright in phase 1. The black box indicates that the learning objective that did not meet the acceptance criteria

**Table 5:** **Finalised curriculum subthemes.** The finalised subthemes and number of objectives in each sub-theme

Sub-Theme	Number of objectives in sub-theme
1. Physical Activity and Human Health	4
2. Medical Issues Related to Exercise	10
3. Injuries Related to SEM	10
4. Basic Science in SEM	7
5. Clinical Pharmacology	2
6. Antidoping	1
7. Sport Team and Event Management	9
8. Specific Groups in SEM	3
9. Intrinsic Skills of a SEM Physician	2
**Total**	**48**

The finalised curriculum can be found in the supplementary material.

## Discussion

This study has utilised Delphi methodology to create an SEM curriculum for undergraduate medical education. The Delphi panel consisted of a broad range of practitioners, with the majority having experience of teaching undergraduate medical student. The final curriculum was made up of 48 LOs.

## Physical activity LOs in the proposed curriculum

The initial proposed curriculum contained 11 objectives relating to medical issues related to exercise, of which 10 were accepted by the expert panel. The one LO that was not accepted related to knowledge of haematological changes in response to exercise (72% acceptance). This was deemed too specific and not at the right level for undergraduate learners. Four LOs were also included in the separate category of physical activity and human health, covering LOs related to physical activity guidelines, common barriers to physical activity and recognising useful physical activity resources. Physical activity reduces the incidence of non-communicable disease (NCDs) such as diabetes mellitus, ischaemic heart disease, several cancer types, and has therapeutic effects in multiple conditions such as musculoskeletal pain, risk of falls and chronic obstructive pulmonary disease (COPD). [30] Despite this, clinicians are often not confident in advising and prescribing exercise interventions. [31] Multiple barriers were found such as fear of exacerbating the condition, insufficient knowledge on which types of exercise are the most beneficial for their patient group, and contraindications. Recent evidence also suggests that medical students themselves appreciate that physical activity is important in preventing disease, but they do not feel confident in physical activity guidelines and would like more teaching on this topic. [11,32,33] There has been a push to better incorporate exercise medicine into undergraduate healthcare curricula, and research into how best to do this. [4,32,34] The findings of this study support the embedding of exercise medicine into undergraduate medical curricula and highlights key Los that should be covered.

## Global application

8 out of the 9 subthemes in the present study were also included in the medical specialty syllabus designed by the international syllabus in Sport and Exercise medicine Group (ISSEMG) [2]. This consensus included a total of 11 subthemes. The differences are likely explained by the undergraduate focus in this study. The USA and Middle Eastern studies also included many overlapping undergraduate themes including physical activity and human health, antidoping and specific groups in SEM. [9,10]

## Ensuring suitability for undergraduate level

Of interest, many LOs were rejected in the first phase of the study due to being topics deemed too SEM-specific for undergraduate curricula, with comments made that they were more appropriate for postgraduate level. In addition, the vast majority of LOs accepted in the final curriculum were in either levels 1 or 2 of Bloom’s taxonomy. This suggests that lower levels of Blooms taxonomy appear to be more appropriate for undergraduate level in sport and exercise medicine curricula.

## Overlap between other specialties

SEM-related topics extend into numerous specialties already well established in the undergraduate medical curricula such as: orthopaedics, rheumatology, and public health. Many of the LOs included in the finalised curriculum produced by this study are likely already covered in medical curricula. For example, many of the 10 LOs in the injuries related to SEM category such as ‘outline common upper limb injuries’ and ‘recognise the indications for common radiological investigations’ are likely to overlap with orthopaedic and musculoskeletal modules already incorporated into medical school education. Similarly, the LOs in the intrinsic skills of an SEM physicians including demonstrating skills such as communication, collaboration and describing the importance of a multi-disciplinary team approach are already covered in medical curriculum.

## Implementation into established undergraduate medical curricula

Whilst this study attempts to map the components of SEM onto a curriculum, actually placing these onto existing curricula remains challenging. The General Medical Council (GMC), who set the core curriculum objectives for medical students to learn during their training, mention SEM-related topics in a number of LOs in “Outcomes for Graduates”. [7] However, medical schools have relative autonomy on the timing and level of depth required for its graduates. Previous reviews of United Kingdom (UK) medical schools have suggested that a proportion of medical schools have not been able to demonstrate evidence of exercise medicine teaching across the core curricula. [5].

While the overlap with other specialties will make embedding this suggested curriculum easier, it is appreciated that there are barriers due to limited space in already crowded medical curricula. Following this study, suggested next steps are to discuss this curriculum with individuals involved in creating, implementing and teaching undergraduate medical curricula to identify feasibility and practical steps for adoption.

## Strengths and Limitations

Our panel consisted of qualified and experienced professionals from relevant backgrounds. The majority of our panel were senior clinicians. In addition, between reviews there was a high response rate, representing a good internal validity. The retention rate between Phase 1 and Phase 2 was above 75%, which improves the reliability of the study. One main limitation was that, due to SEM being a relatively small, albeit growing, speciality, the vast majority of our panel were highly interested in SEM, thereby introducing potential selection bias.

## Conclusion

Sport and Exercise Medicine is a broad ranging and rapidly growing speciality, with significant importance in tackling the burden against NCDs. It is important to establish SEM education in all levels of medical education, including undergraduate level. To our knowledge, this is the first published version of a Delphi SEM curriculum for undergraduate medical teaching. Future work should explore the opinions of individuals working in medical education, and those whose primary focus is not in SEM, to discuss opinions and how it could be best implemented into medical school curricula. In addition, it would be advantageous to compare this curriculum with undergraduate SEM curriculums used in other countries around the world.

## Electronic supplementary material

Below is the link to the electronic supplementary material.

Supplementary Material 1

## Data Availability

The data for this study is stored on Hull York Medical School servers and is not publicly available. Data can be made available upon reasonable request to the corresponding author.

## Declarations

### Authors’ information

Twitter: @DaneVishnubala @adliqbal @krmarino1 @pandyatej @drdavidsalman @andypringle6 @DrDaveEastwood @gabs_finn.

## List of Abbreviations

SEM: Sports and Exercise Medicine
UK: United Kingdom
LOs: Learning objectives
USEMS: Undergraduate Sport and Exercise Medicine Society
BASEM: British Association of Sport and Exercise Medicine
FSEM: Faculty of Sport and Exercise Medicine
MFSEM: Membership of the FSEM
FFSEM: Fellowship of the FSEM
