# Supplementary material for: Creating a sport and exercise medicine undergraduate syllabus: a delphi study
Source: BMC Med Educ. 2023 Mar 23;23:179. doi: 10.1186/s12909-023-04139-x (PMC10035170; doi:10.1186/s12909-023-04139-x)
Supplement: Supplementary file 1 — Supplementary Material 1 [file 12909_2023_4139_MOESM1_ESM.docx]

| Theme | Learning Objective |
| --- | --- |
| 1. Physical Activity and Human Health | Outline the role of physical activity in the prevention and treatment of disease |
|  | Outline the current UK CMO physical activity guidelines and recommendations for all age groups, disabled adults, pregnant women and women after childbirth |
|  | Discuss common barriers to physical activity: environmental, social, physical and psychological |
|  | Recognise key national and international physical activity resources available to patients and clinicians |
| 2. Medical Issues Related to Exercise | Discuss physical activity benefits and considerations in patients with the following respiratory conditions: COPD Asthma |
|  | Discuss physical activity benefits and considerations in patients with the following cardiovascular conditions: Atrial Fibrillation Hypertension Ischaemic heart disease |
|  | Describe common gastrointestinal issues in relation to physical activity |
|  | Discuss physical activity benefits and considerations in patients with the following metabolic issues in relation to: Diabetes Thyroid disease Obesity |
|  | Recognise the impact of physical activity on the immune system |
|  | Discuss physical activity benefits and considerations in patients with the following rheumatological conditions: Inflammatory Arthritis Osteoarthritis Fibromyalgia and chronic pain Connective tissue disorders Hypermobility syndromes Osteoporosis |
|  | Outline physical activity benefits and considerations in patients with psychological and mental health conditions |
|  | Discuss physical activity benefits and considerations in patients with the following adult musculoskeletal issues: Chronic pain Lower back pain Chronic musculoskeletal issues |
|  | Identify common adult musculoskeletal conditions |
|  | Outline common paediatric musculoskeletal issues |
| 3. Injuries Related to SEM | Outline the principles of prevention, diagnosis, treatment and rehabilitation of common injuries related to sports and physical activity |
|  | Outline the principles of tissue injury and repair in the musculoskeletal system |
|  | Outline common head and neck injuries |
|  | Outline common upper limb injuries |
|  | Outline common trunk, abdominal and thoracic spinal injuries |
|  | Outline common lumbar spine and pelvic injuries |
|  | Outline common lower limb injuries |
|  | Recognise the indications for common radiological investigations |
|  | Describe the role of physical activity in rehabilitation |
|  | Appreciate the role of orthotics, splinting, bracing and taping |
| 4. Basic Science in SEM | Outline the physiological effects of physical activity on the human body |
|  | Recognise the role of sports psychology in sport and physical activity |
|  | Describe clinically relevant regional musculoskeletal anatomy |
|  | Relate musculoskeletal anatomical knowledge to common conditions and presentations |
|  | Outline the various sources of energy available to the body |
|  | Describe cellular metabolism and biomechanical pathways of energy production |
|  | Describe appropriate nutrition for a healthy lifestyle |
| 5. Clinical Pharmacology | Outline important considerations when prescribing safely |
|  | Outline the pharmacological management of acute pain in musculoskeletal conditions in sport and physical activity |
| 6. Antidoping | Be aware that certain medications are prohibited in sport |
| 7. Sport Team and Event Management | Outline the roles and responsibilities of the SEM physician in the team environment |
|  | Discuss the importance of medical screening |
|  | Demonstrate basic life support in a simulated environment |
|  | Discuss the causes of cardiac and traumatic sudden death in sport |
|  | Discuss the clinical features of concussion and head injury in relation to sports and physical activity |
|  | Discuss the clinical features of common acute musculoskeletal and soft tissue injuries in relation to sports and physical activity |
|  | Recognise the acutely unwell patient |
|  | Recognise the role of pre-hospital care in sport and physical activity |
|  | Discuss the basic management of common fractures and dislocations in sports and physical activity |
| 8. Specific Groups in SEM | Outline the effects that the ageing process can have on an elderly individual’s ability to safely take part in sport and physical activity |
|  | Outline the effects that pregnancy can have on an individual’s ability to safely take part in sport and physical activity |
|  | Describe social, psychological, religious and cultural factors that influence physical activity participation |
| 9. Intrinsic Skills of a SEM Physician | Demonstrate the following skills commonly used in practice:  Communication - Collaboration - Leadership and management - Health advocacy - Safety - Research - Teaching - Learning - Professionalism - Consideration of ethics - Awareness of health inequalities |
|  | Describe the importance of a multi-disciplinary team approach |
